# Supplementary material for: Cultivating river sediments into efficient denitrifying sludge for treating municipal wastewater
Source: R Soc Open Sci. 2019 Sep 25;6(9):190304. doi: 10.1098/rsos.190304 (PMC6774965; doi:10.1098/rsos.190304)
Supplement: Fig.2. Changes of nitrogen form during cultivation process in effluent and influent [file rsos190304supp1.pdf]

Raw data of Fig.2. Changes of nitrogen form during cultivation process in effluent and influent.

Time (h)	NO_3^- in the influent(mg/L)	NO_3^- in the effluent(mg/L)	NO_3^- removal rate（%）	NO_2^-in the effluent(mg/L)
0	19.89	18.72	5.88235	0
24	20.21	19.35	4.25532	0
48	19.25	17.01	11.63636	0
72	19.36	16.38	15.39256	0
96	20.73	17.86	13.84467	0
120	19.24	16.72	13.09771	0
144	20.29	17.13	15.57417	0
168	20.44	13.25	35.17613	0
192	20.76	9.16	55.87669	0
216	20.04	6.25	68.81238	0
240	20.12	5.65	71.91849	0
264	19.48	5.08	73.92197	0
288	19.31	3.39	82.44433	0
312	19.69	4.17	78.82174	0
336	19.75	3.22	83.6962	0
348	19.56	3.03	84.5092	0
360	20.28	2.95	85.45365	0
372	19.76	2.9	85.32389	0
384	20.09	2.87	85.71429	0
396	19.55	2.65	86.44501	0
408	20.03	2.67	86.67	0
420	20.12	2.75	86.33201	0
432	19.81	2.69	86.421	0
444	20.36	2.74	86.54224	0
452	50.08	41.03	18.07109	0
460	50.63	40.59	19.83331	0
468	49.64	38.31	22.82434	0
476	50.52	39.88	21.07346	0
484	49.71	37.14	25.28967	0.45
492	49.64	36.92	25.63648	0.51
500	50.29	35.2	30.01432	0.56
508	50.16	34.45	31.33073	0.76
516	50.02	31.68	36.6704	0.85
524	50.4	33.25	34.02778	0.9
532	49.56	30.81	37.84296	1.34
540	49.59	29.88	39.74835	1.56
548	49.56	31.33	36.7837	3.67
556	49.5	27.39	44.67114	4.56
564	49.62	26.55	46.49766	5.23
572	49.48	25.2	49.07033	6.33
580	50.41	24.17	52.05887	6.15
588	50.56	24.34	51.87441	5.34
596	49.61	21.6	56.46566	5.3
604	49.992	20.73	58.53337	5.65
612	50.32	19.52	61.20827	4.75
620	50.648	19.05	62.38746	5.21
628	50.08	17.56	64.9361	5.19
636	50.696	18.55	63.40934	5.73
644	49.976	20.67	58.64015	5.68
652	49.584	18.39	62.91142	4.78
660	49.96	15.53	68.91513	4.43
668	50.72	13.89	72.61435	4.15
676	49.64	12.56	74.69782	3.87
684	50.104	11.01	78.02571	3.75
692	49.488	10.37	79.04543	2.13
700	50.072	10.06	79.90893	2.76

708	49.344	9.93	79.87597	3.22
716	49.36	9.68	80.38898	2.95
724	50.752	9.45	81.38004	2.99
732	49.784	9.36	81.19878	3.35
740	49.544	9.35	81.12789	3.19
748	49.84	9.23	81.48074	2.98
756	50.792	9.06	82.16255	3.28
764	50.688	9.15	81.94839	3.22
772	50.72	9.22	81.82177	2.78
780	49.944	9.55	80.87858	2.93
788	50.576	9.32	81.57229	3.21
796	50.016	9.08	81.84581	3.25
804	50.368	9.53	81.07926	2.91
810	50.176	18.16	63.8074	1.96
816	49.36	18.35	62.82415	2.08
822	49.888	18.08	63.75882	2.22
828	50.736	18.27	63.99007	1.97
834	50.352	18.12	64.01335	1.98
840	49.488	17.25	65.14306	3.77
846	49.232	15.89	67.72424	6.42
852	49.576	15.77	68.19025	5.98
858	49.712	16.1	67.61345	6.24
864	49.304	15.22	69.13029	6
870	50.216	14.73	70.66672	5.55
876	49.68	14.22	71.37681	5.19
882	50.528	15.3	69.71976	5.32
888	50.056	13.88	72.27106	6.09
894	49.56	13.25	73.26473	5.76
900	50.328	12.67	74.82515	5.14
906	49.272	12.92	73.77821	5.35
912	50.328	12.66	74.84502	5.28
918	49.544	12.53	74.70935	5.93
924	49.376	12.41	74.86633	4.78
930	49.872	12.82	74.29419	4.22
936	49.224	11.79	76.04827	4.31
942	49.568	11.65	76.49693	4.29
948	49.576	11.55	76.70244	4.16
954	49.752	13.33	73.20711	4.28
960	49.968	12.08	75.82453	5.05
966	49.488	11.7	76.3579	4.75
972	50.144	11.55	76.96634	4.2
978	49.936	11.34	77.29093	5.37
984	49.752	10.88	78.13153	5.03
990	49.208	11.25	77.13786	4.14
996	49.704	11.09	77.68791	3.28
1002	50.288	12.25	75.64031	2.97
1008	49.472	11.38	76.99709	1.03
1014	49.776	11.36	77.17776	2.04
1020	49.736	11.19	77.50121	1.25
1026	50.224	12.04	76.0274	1.76
1032	49.752	11.08	77.72954	1.44
1038	50.336	11.25	77.65019	2.35
1044	50.432	11.31	77.57376	1.89
1048	50.016	19.65	60.71257	1.65
1052	50.6	19.49	61.48221	1.34
1056	50.264	18.22	63.75139	2.88
1060	50.68	16.45	67.54144	1.69
1064	50.192	15.33	69.45728	2.01

1068	50.128	14.78	70.51548	2.32
1072	50.168	18.76	62.60565	1.35
1076	49.856	17.24	65.42041	0.97
1080	50.656	15.36	69.67783	0
1084	49.48	13.19	73.34276	0
1088	49.272	11.09	77.49229	1.66
1092	49.648	13.28	73.25169	2.14
1096	50.488	15.28	69.73538	1.79
1100	50.376	13.65	72.90376	1.53
1104	49.832	12.07	75.77862	1.1
1108	50.624	11.21	77.85635	1.42
1112	49.736	11.09	77.70227	1.29
1116	50.136	11	78.05968	1.18
1120	49.704	13.13	73.58362	1.65
1124	49.536	11.03	77.73337	1.22
1128	49.864	10.25	79.44409	1.3
1132	50.016	9.78	80.44626	1.24
1136	49.576	8.35	83.15717	1.15
1140	49.48	7.57	84.70089	1.08
1144	49.768	10.29	79.32406	1.29
1148	50.4	8.45	83.23413	0.88
1152	50.032	7.19	85.6292	0.98
1156	49.392	7.23	85.362	1.66
1160	49.728	7.09	85.74244	2.14
1164	50.04	7.22	85.57154	1.44
1168	50.248	7.27	85.53176	1.29
1176	50.224	7.18	85.70405	1.18
1184	49.696	7.35	85.21008	1.35
1192	50.112	7.29	85.45259	1.66
1200	49.48	7.81	84.21584	2.14
1208	50.112	7.39	85.25303	1.59
1216	49.912	7.03	85.91521	2.14
1224	49.304	7.22	85.35616	1.2
1232	49.464	7.17	85.50461	1.99
1240	49.48	7.2	85.44867	1.25
1248	49.528	7.11	85.64448	1.72
1256	50.24	7.03	86.00717	1.13
1264	49.256	6.28	87.25028	1.27
1272	49.744	7.32	85.28466	0.77
1280	49.704	7.19	85.53436	1.35
1288	49.24	7.2	85.37774	0.96
1296	49.376	6.42	86.99773	1.18
1304	49.704	6.66	86.60068	0.55
1312	50.696	7.95	84.31829	1.23
1320	50.144	7.32	85.40204	1.17
1328	49.256	8.26	83.23047	1.2
