# Supplementary material for: Cultivating river sediments into efficient denitrifying sludge for treating municipal wastewater
Source: R Soc Open Sci. 2019 Sep 25;6(9):190304. doi: 10.1098/rsos.190304 (PMC6774965; doi:10.1098/rsos.190304)
Supplement: Fig.3. Response surface [file rsos190304supp2.pdf]

Raw Data of Fig.3 Response Surface Analysis

Hydraulic retention time (HRT， h)	Sediment addition (SA, ml/L)	Nitrate concentration (NC, mg/L)	Nitrate removal rate (NRR, %)
4	6	20	77.72
6	4	20	76.69
6	8	20	80.18
8	6	20	85.91
4	4	50	77.65
4	8	50	80.18
6	6	50	85.5
8	4	50	83.14
8	8	50	85.75
4	6	80	77.73
6	4	80	80.38
6	8	80	83.07
8	6	80	86.25
