# Supplementary material for: Cultivating river sediments into efficient denitrifying sludge for treating municipal wastewater
Source: R Soc Open Sci. 2019 Sep 25;6(9):190304. doi: 10.1098/rsos.190304 (PMC6774965; doi:10.1098/rsos.190304)
Supplement: Fig.5 High-throughput sequencing analysis of the sediment after cultivation [file rsos190304supp3.pdf]

Raw Data of Fig.5 High-throughput sequencing analysis of the sediment after cultivation

genera	the relative abundance
Diaphorobacter	33.82%
Dechloromonas	0.47%
Paracoccus	24.49%
Pleomorphomonas	0.49%
unclassified	5.08%
Trichococcus	0.53%
Thauera	3.85%
Ignavibacterium	0.64%
Cloacibacterium	2.85%
Rhizobium	0.68%
Aquimonas	2.43%
Simplicispira	0.82%
Chiayiivirga	2.14%
Pseudoxanthomonas	1.06%
Flavobacterium	2.13%
Stenotrophomonas	1.43%
Chryseobacterium	1.84%
Petrimonas	1.45%
Brevundimonas	1.84%
Anaerorhabdus	1.45%
Other	10.52%
