# Supplementary material for: Cultivating river sediments into efficient denitrifying sludge for treating municipal wastewater
Source: R Soc Open Sci. 2019 Sep 25;6(9):190304. doi: 10.1098/rsos.190304 (PMC6774965; doi:10.1098/rsos.190304)
Supplement: Fig.6. Changes of NO3--N in municipal wastewater treatment [file rsos190304supp4.pdf]

Raw Data of Fig.6 Changes of NO_3^--N in municipal wastewater treatment

Time (d)	NO_3^-concentration in influent (mg/L)	Influent error	NO_3^-concentration in effluent (mg/L)	Effluent error	NO_3^-removal rate %
1	23.15	0.75	19.3	1.86	16.63067
2	25.35	0.64	20.55	2.28	18.93491
3	20.5	0.22	16.35	1.74	20.2439
4	22.55	0.53	17.55	3.34	22.17295
5	23.5	1.81	16.3	1.96	30.6383
6	22.75	0.53	13.35	2.62	41.31868
7	21.25	1.32	12.15	1.44	42.82353
8	21.25	0.87	12.55	2.66	40.94118
9	22.44	0.68	13.8	1.31	38.50267
10	24.22	1.96	12.7	2.91	47.564
11	25.45	0.45	10.95	2.12	56.97446
12	26.6	1.36	8.85	1.97	66.72932
13	20.65	1.74	5.75	2.58	72.15496
14	22.75	0.81	4.95	1.21	78.24176
15	23.05	0.87	3.51	2.16	84.77223
16	26.37	0.19	3.73	1.83	85.85514
17	24.55	1.82	21.95	1.13	10.59063
18	23.45	2.27	19.06	3.47	18.72068
19	25.5	0.45	15.12	1.55	40.70588
20	27.95	2.14	14.17	1.94	49.30233
21	26.13	0.95	10.08	2.67	61.42365
22	25.08	1.97	7.55	1.89	69.89633
23	23.27	0.87	6.32	3.37	72.84057
24	26.59	2.46	5.28	1.36	80.14291
25	25.32	0.68	5.17	2.5	79.58136
26	24.09	1.97	4.28	2.68	82.23329
27	26.8	1.17	5.05	1.2	81.15672
28	21.45	2.25	5.1	2.32	76.22378
29	21.3	1.14	4.9	2.62	76.99531
30	22.65	2.58	5.35	1.98	76.37969
31	23.43	0.83	4.55	0.64	80.58045
32	28.2	1.93	5.03	2.15	82.16312
33	20.93	2.61	4.11	0.76	80.36312
34	26.65	2.36	3.9	2.76	85.36585
35	29.45	2.74	3.74	2.01	87.30051
36	25.75	2.25	4.6	0.84	82.13592
37	24.65	0.88	4.27	2.67	82.67748
38	21.91	2.7	4.25	1.87	80.60246
39	23.56	1.24	4.64	2.36	80.3056
